# TCR-like antibodies in cancer immunotherapy

**DOI:** 10.1186/s13045-019-0788-4

**Published:** 2019-09-14

**Authors:** Qinghua He, Zhaoyu Liu, Zhihua Liu, Yuxiong Lai, Xinke Zhou, Jinsheng Weng

**Affiliations:** 1 Department of Center Laboratory, The Fifth Affiliated Hospital of Guangzhou Medical University, Guangzhou, 510700 China; 2 Department of Lymphoma and Myeloma, Division of Cancer Medicine, The University of Texas MD Anderson Cancer Center, 1414 Holcombe Boulevard, Houston, TX 77030 USA

**Keywords:** T cell receptor, TCR-like antibody, Antibody, Tumor antigen, Immunotherapy

## Abstract

Cancer immunotherapy has been regarded as the most significant scientific breakthrough of 2013, and antibody therapy is at the core of this breakthrough. Despite significant success achieved in recent years, it is still difficult to target intracellular antigens of tumor cells with traditional antibodies, and novel therapeutic strategies are needed. T cell receptor (TCR)-like antibodies comprise a novel family of antibodies that can recognize peptide/MHC complexes on tumor cell surfaces. TCR-like antibodies can execute specific and significant anti-tumor immunity through several distinct molecular mechanisms, and the success of this type of antibody therapy in melanoma, leukemia, and breast, colon, and prostate tumor models has excited researchers in the immunotherapy field. Here, we summarize the generation strategy, function, and molecular mechanisms of TCR-like antibodies described in publications, focusing on the most significant discoveries.

## Background

Cancer immunotherapy has been cited as the greatest scientific breakthrough of 2013 [1]. The core element of this success is antibody therapy. In the last 40 years, more than 74 different antibody-based molecules have been approved for use in clinical treatment in the European Union, the USA, and Japan [2, 3]. Currently, there are more than 864 antibodies in phase I, II, or III clinical trials, covering a wide spectrum of diseases in the human body [3]. These have demonstrated the powerful and specific effects of antibody therapy in the field of human diseases and prompt us to seek further breakthroughs in this field.

Activated memory plasma cells secrete antibodies that consist of an fragment antigen-binding (Fab) and a fragment crystallizable region (Fc). After binding to the antigen through their highly variable Fab regions, the antibodies can mediate anti-tumor effects through many different mechanisms. Herceptin, the anti-human epidermal growth factor receptor 2 (HER2) antibody, can bind directly to breast cancer cells and inhibit their metastasis through the induction of apoptosis [4]; rituximab, the anti-cluster of differentiation 20 (CD20) chimeric antibody, can induce lymphoma cell death through antibody-dependent cellular cytotoxicity (ADCC) or complement-dependent cytotoxicity (CDC) [5]; Opdivo (nivolumab, anti-PD-1), Keytruda (pembrolizumab, anti-PD-1), Yervoy (ipilimumab, anti-CTLA-4), and Kymriah (tisagenlecleucel, anti-CD19 CAR-T cells) can induce tumor lysis through immune cell activation and recruitment [3]; and Mylotarg (gemtuzumab ozogamicin, anti-CD33 antibody-drug conjugate), Adcetris (brentuximab vedotin, anti-CD30 antibody-drug conjugate), and Kadcyla (ado-trastuzumab emtansine, anti-HER2 antibody-drug conjugate) can induce tumor death through conjugated cytotoxin delivery [6]. All of these have attested to the dramatic effects of antibody therapy against cancer cells. However, one limitation of traditional antibody therapy is that the antibodies can target only cell surface antigens and have no effect on intracellular proteins.

**Fig. 1:**
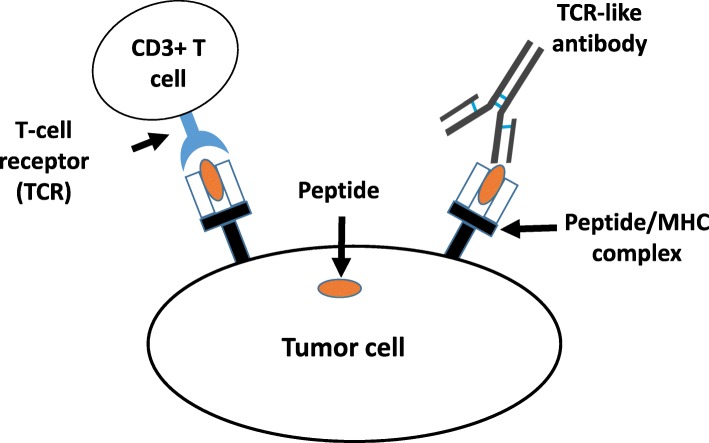
Schematics of T cell receptor (TCR) and TCR-like receptor. Both TCR and TCR-like antibodies recognize the peptide/MHC complex on the surface of tumor cells

Most tumor-specific antigens that control cell growth, proliferation, and death are intracellular. To target these antigens, a specific group of antibodies called T cell receptor (TCR)-like/mimic antibodies has been developed for clinical therapy [7]. The intracellular tumor-specific antigens can go through the major histocompatibility complex (MHC) class I signaling pathway and present as tumor-specific peptide/MHC complexes on the tumor cell surfaces [8]. TCR-like antibodies recognize the peptide/MHC complexes on the tumor cell surfaces in the same manner as authentic TCRs (Fig. 1). The recognition of the peptide/MHC complex by TCRs expressed on the surface of T cells can trigger various effects, such as T cell proliferation and differentiation and cytokine or chemokine secretion [9]. The recognition of the peptide/MHC complex by TCR-like antibodies, however, can trigger much broader pharmacological pathways than that of the TCRs in T cells [7]. TCR-like antibodies can trigger ADCC, CDC, antibody-dependent cellular phagocytosis (ADCP), or the direct induction of apoptosis [10]. In addition, TCR-like antibodies can be converted to a chimeric antigen receptor (CAR) structure to mediate the specific recognition of tumor cells by T cells, such as CAR-T cells [11].

Soluble TCRs have proven to be difficult to engineer in vitro, and their inherently low affinity for their targets limits their use as a single molecular tool to detect the expression of the peptide/MHC complex on the tumor cell surface [12–14]. To overcome these limitations, TCR-like antibodies have been developed as an in vitro tool. For example, TCR-like antibodies have been conjugated with fluorescent reagents to detect the expression level of the Wilms tumor 1 (WT1) RMFPNAPYL peptide/human leukocyte antigen (HLA)-A2 complex on the leukemia cell surface, which offers a clear map of the tumor-specific antigen profile [15]. TCR-like antibodies can also be conjugated with cytotoxic organic compounds, such as antibody-drug conjugates (ADCs), radionuclides, and protein toxins, to mediate the specific killing of tumor cells [16]. Furthermore, immunomodulators or secondary antibodies can be conjugated with the TCR-like antibodies to mediate specific immune responses around the tumor site, as in bi-specific T cell engagers (BiTE) [17]. Finally, in comparison with the tedious process of preparing a large number of tumor antigen-specific T cells for each patient, as in CAR-T or TCR-T therapy, TCR-like antibodies can be prepared in large amounts, stored for long periods of time, and used as off-the-shelf products for patients, significantly reducing clinical costs. Hence, the research on TCR-like antibodies in the field of tumor therapy has exploded over the last decades [10, 18, 19].

There are currently more than 40 TCR-like antibodies in pre-clinical development, and most of them show strong anti-tumor effects both in vitro and in vivo (Table 1). Here, we summarize the most significant discoveries for TCR-like antibodies, including the antigen selection, generation strategy, function, and molecular mechanisms of TCR-like antibodies, the advantages and disadvantages of TCR-like antibodies versus other immunotherapies, and future directions for TCR-like antibody development.

**Table 1:** TCR-like antibodies in human diseases. Information of published TCR-like antibodies was collected from literature and reference [18] with the consent of the authors

Antigen	Epitope sequence	MHC allele	Diseases targeted	Function	TCR-like antibody format	Clone	Generation strategy	Reference
MAGE1	EADPTGHSY	HLA-A*0101	Melanoma	Detection	Fab	Fab-G8	Phage	[20]
	EADPTGHSY	HLA-A*0101	Melanoma	CAR-T	Fab	Fab-G8	Phage	[21]
	EADPTGHSY	HLA-A*0101	Melanoma	CAR-T	Fab	Fab-G8/Fab-Hyb3	Phage	[22]
GP100	KTWGQYWQV	HLA-A*0201	Melanoma	Detection	Fab	G2D12, G3G4	Phage	[23, 24]
	IMDQVPFSV	HLA-A*0201	Melanoma	Detection	Fab	1A9, 1C8, 1A11, 1A7	Phage	[23, 24]
	YLEPGPVTV/A	HLA-A*0201	Melanoma	detection/inhibition	Fab	2F1, 2B2, 2C5, 2D1	Phage	[23, 24]
	IMDQVPFSV	HLA-A*0201	Melanoma	Immunotoxin	scFv-PE38	G1	Phage	[16]
	ITDQVPFSV	HLA-A*0201	Melanoma	CAR-T	sdAb-CAR	GPA7	Phage	[25]
	LLLTVLTVL	HLA-A*0201	Melanoma	Immunotoxin	Fab	2F1-PE38KDEL	Phage	[26]
hTERT	ILAKFLHWL	HLA-A*0201	Melanoma, prostate cancer	Detection/inhibition	Fab	4A9, 4G9	Phage	[27]
	RLVDDFLLV	HLA-A*0201	Melanoma, prostate cancer	Detection/inhibition	Fab	3H2, 3G3	Phage	[27]
MUC1	LLLTVLTVV	HLA-A*0201	Breast cancer	Detection	Fab	M2B1, M2F5, M3A1, M3B8, M3C8	Phage	[28]
NY-ESO-1	SLIMWITQC	HLA-A*0201	Melanoma	Detection/inhibition	Fab	3M4E5;3M4F4;T1	Phage	[29]
MAGE3	FLWGPRALV	HLA-A*0201	Melanoma	Detection	mIgG1	7D4, 8A11, 2G12, 9E6	Hybridoma	[30]
hCGβ	GVLPALPQV	HLA-A*0201	Ovarian, colon, breast cancer	CDC, ADCC, direct	mIgG2a	RL4B/3.2G1	Hybridoma	[31]
	GVLPALPQV	HLA-A*0201	Ovarian, colon, breast cancer	Detection	IgG1	1B10	Hybridoma	[21]
	TMTRVLQGV	HLA-A*0201	Ovarian, colon, breast cancer	Detection	IgG1	3F9	Hybridoma	[32]
Her2/Neu	KIFGSLAFL	HLA-A*0201	Breast, colon cancer	Detection/inhibition	IgG1	1B8	Hybridoma	[33]
Melan-A/MART-1	EAAGIGILTV	HLA-A*0201	Melanoma	Detection	Fab	2M3F11;3N4E9;2N4B4;E6;H4	Phage	[34]
	EAAGIGILTV	HLA-A*0201	Melanoma	Immunotoxin	Fab-PE38	CAG10, CLA12	Phage	[26]
TARP	FLRNFSLML	HLA-A*0201	Breast, prostate cancer	Immunotoxin	Fab-PE38	Fab-D2	Phage	[35]
p53	RMPEAAPPV	HLA-A*0201	Various tumors, breast cancer	ADCC, ADCP, CDC	IgG1	T1-116C	Hybridoma	[36]
	RMPEAAPPV	HLA-A*0201	–	Detection	IgG1, IgG2b	T1-29D, T1-84C	Hybridoma	[37]
	GLAPPQHLIRV	HLA-A*0201	–	Detection	IgG1, IgG2a, IgG1	T2-108A, T2-2A, T2-116A	Hybridoma	[37]
Tyrosinase	YMDGTMSQV	HLA-A*0201	Melanoma	Detection	Fab	TA2	Phage	[38]
p68	YLLPAIVHI	HLA-A*0201	Breast cancer	ADCP/direct	mIgG2a	RL6A	Hybridoma	[39]
MIF	FLSELTQQL	HLA-A*0201	Breast cancer	CDC, ADCC, direct	IgG2a	RL21A	Hybridoma	[40]
Proteinase 3	VLQELNVTV	HLA-A*0201	AML	CDC, CAR-T	IgG2a	8F4	Hybridoma	[41, 42]
WT1	RMFPNAPYL	HLA-A*0201	Leukemia, ovarian, colon cancer	ADCC, ADCP	hIgG1	ESK1	Phage	[15]
	RMFPNAPYL	HLA-A*0201	Leukemia	CAR-T	Fab	F2, F3	Phage	[43]
	RMFPNAPYL	HLA-A*0201	Leukemia	ADCC, CAR-T	scFv	Clone45	Phage	[44]
HA-1H	VLHDDLLEA	HLA-A*0201	Leukemia	CAR-T	scFv, scFv-CAR	#131	Phage	[11]
PRAME	ALYVDSLFFL	HLA-A*0201	Leukemia, lymphoma	ADCC, CDC, ADCP	hIgG1	Pr20	Phage	[45]
HTLV-1(TAX-11)	LLFGYPVYV	HLA-A*0201	T-Cell leukemia/lymphoma	Detection	Fab	T3A4,T3D4;T3F2;T3E3;T3D3;T2H9	Phage	[46, 47]
Influenza(M1-58)	GILGFVFTL	HLA-A*0201	Flu	Detection	Fab	M1-D1,M1-G8;M1-D12;M1-A2	Phage	[48]
HBV (ENV-183)	FLLTRILTI	HLA-A*0201	Hepatitis B	Detection/intracellular delivery of cargo	mIgG1	N/A	Hybridoma	[49]
HIV-1 (Nef-105)	RRQDILWIY	HLA-C*07	AIDS	Surface co-expression with fas-ligand on virion particle	Fab	C3	Phage	[50]
HIV-1 (elf4G-720)	VLMTEDIKL	HLA-A*0201	AIDS	Detection	mIgG1	4F7	Hybridoma	[51]
HIV-1 (Nef-138)	RYPLTFGWCF	HLA-A*2401	AIDS	Detection	Fab	scFv#3, scFv#27	Phage	[52]
CMV (pp65-495)	NLVPMVATV	HLA-A*0201	Mucoepidermoid carcinoma	Detection	Fab	H9	Phage	[53]

### Tumor antigen selection

Tumor antigens are grouped into several categories according to their origins and specificity. The first category is oncovirus antigens, which include Epstein-Barr nuclear antigen 1-3 (EBNA 1-3), latent membrane protein 1 (LMP1), and LMP2 derived from Epstein-Barr virus (EBV) [54], hepatitis B virus X protein (HBX) from hepatitis B virus (HBV) [55, 56], core nonstructural protein 3 (NS3) and nonstructural protein 5A (NS5A) from hepatitis C virus (HCV) [57], type E5, E6, and E7 proteins from human papillomavirus (HPV) [58], viral transactivator (Tax) from human T cell leukemia-lymphoma virus (HTLV) [59], latency-associated nuclear antigen (LANA), virus active G protein-coupled receptor homolog (vGPCR), and virus IFN-inducible factor (vIRF-1) from Kaposi sarcoma-associated herpesvirus (KSHV) [60], structural protein PP65 from cytomegalovirus (CMV) [61], and group-specific antigen (gag) and pol reading frame 468 (Pol468) from human immunodeficiency virus (HIV) [62]. The oncoviruses can cause many diseases, including Burkitt’s lymphoma (BL), non-Hodgkin’s B cell lymphoma (NHL), nasopharyngeal carcinoma (NPC), hepatocellular carcinoma (HCC), cervical cancer, adult T cell leukemia (ATL), primary effusion lymphoma (PEL), Kaposi’s sarcoma (KS), and Merkel cell carcinoma (MCC). The oncovirus antigens are highly tumor-specific, as they are unique to the oncoviruses and are not shared by normal human tissues. However, viral infections cause only about 10–15% of all human cancers, and some healthy individuals do not develop cancer even with the infection of an oncovirus [60, 63, 64]. Hence, the oncovirus antigens are of limited use in the clinic.

The second group of tumor antigens involves chromosome/gene mutations in cancer cells [65, 66]. These mutations include chromosomal translocation, loss, duplication, and loss or point mutation of nucleic acids in the exons, introns, or regulatory regions of genes [67]. These mutations can lead to the expression of truncated proteins, fusion proteins, or neoantigens that are unique to cancer cells, such as beta-catenin S37F in melanoma [68], alpha-actinin-4 K122N in lung cancer [69], heat shock protein 70 kilodalton-2 (hsp70-2) F293I in renal cancer [70], Kirsten rat sarcoma viral oncogene (K-ras) G12D in colon cancer [71], myeloid differentiation primary response 88 (MYD88) L265P in hairy cell leukemia [72], and B cell receptor-Abelson murine leukemia viral oncogene homolog 1 (BCR-ABL) fusion protein in chronic myeloid leukemia [73]. These antigens are tumor cell-specific. However, some types of cancer have a high burden of genetic mutations, whereas other types of cancers may not; in addition, many genetic mutations are unique to the tumor cells of individual patients [74, 75]. Hence, this group of tumor antigens is difficult to target with the current adoptive cellular therapy strategy.

The third group of tumor antigens is the cancer-testis antigens, which are overexpressed in multiple types of tumor cells of patients [76, 77]. In healthy donors, this group of antigens is expressed only in immune-privileged organs, such as the testis or placenta. Because the immune-privileged organ cells do not express MHC alleles, TCRs that recognize the peptide/MHC complex derived from this group of antigens will not damage the normal tissue cells [78]. Moreover, high-affinity TCRs targeting cancer-testis antigens can be isolated from the peripheral blood of normal donors because of the absence of cancer-testis antigens in the peripheral blood [79, 80]. Hence, this group of tumor antigens, including New York esophageal squamous cell carcinoma-1 (NY-ESO-1), melanoma-associated antigen A (MAGE-A), and synovial sarcoma X (SSX), comprises the largest number in current clinical trials [81, 82].

The fourth group of tumor antigens involves antigens with minimal or limited expression in normal cells, such as carcinoembryonic antigen (CEA), melanoma antigen recognized by T cells 1 (MART-1), and tyrosine kinase 10 [83–85]. Targeting these antigens can damage normal tissues, and sophisticated technology is needed for the future development of immunotherapy against these antigens [86, 87]. This group also includes antigens derived from non-essential organs, such as CD19 and CD20 from B cells [88]. Targeting these antigens can cause non-fatal damage to normal tissue, which medical interventions can cure [89, 90].

Importantly, about 95% of the aforementioned tumor antigens are intracellular proteins, and very few tumor-specific antigens are extracellular [91]. Thus, to target tumors through tumor-specific antigens, a novel strategy must be developed.

### TCR-like antibody generation

Because intracellular proteins can be digested into small peptides in the proteasome of a cell, which can be conjugated with MHC molecules in the endoplasmic reticulum (ER) and transported to the tumor cell surface, the peptide/MHC complex on the tumor cell surface has been deemed as a tumor-specific antigen [92]. MHC class I molecules are expressed on the surface of all nucleated cells, and numerous studies have demonstrated the feasibility of targeting tumors through the recognition of the peptide/MHC complex on the cell surface [85, 93, 94].

In 1981, Wylie and Klinman conducted the first study of a TCR-like antibody [95]. To study the immune response to influenza, they injected influenza virus and the virus-infected cell line PR8-L929 into C3H/HeJ and BAL6.K mouse strains. They found that approximately one third of the virus-specific antibodies reacted to viral hemagglutinin (HA) or neuraminidase. The remainder of virus-specific antibodies recognized antigens found on the surface of virus-infected PR8-L929 cells but not on the virion or uninfected cells. It was later found that the MHC participated in the recognition of viral antigens by the antibodies [96]. Similar results have been found in mouse cells transformed with simian virus antigen (SV40), murine cytomegalovirus (MCMV) pp89 (168–176) peptides, vesicular stomatic virus (VSV), and EBV [97–99]. It was demonstrated that mouse MHC conformational epitopes are peptide-specific. The monoclonal antibody (mAb) 34.4.20 recognized the VSV nucleoprotein (52–59) peptide on mouse H-2Kb but not ovalbumin (OVA) (257–264), MCMV pp89 (168–176), or influenza nucleoprotein (Y345–360) peptides on the same MHC allele [98]. Although these studies did not test the cytotoxic effect of TCR-like antibodies, they provided clear evidence that TCR-like antibodies generated in the mouse B cells can specifically bind to the peptide/MHC complex on the cell surface.

In 2000, Chames and colleagues reported the first TCR-like antibody targeting human tumor antigens [20]. Using the phage library technique, they isolated a human antibody directed against the EADPTGHSY peptide encoded by *MAGE-A1* and presented by the HLA-A1 molecule. *MAGE-1* is a cancer-testis gene overexpressed in multiple cancers but with restricted expression in the testis of a healthy person [100]. The phage Fab antibody bound to the HLA-A1 molecule complexed with the MAGE-A1 peptide but not to the HLA-A1 molecule complexed with other peptides, indicating the specificity of the antibody. Furthermore, the TCR-like antibody bound to the *MAGE-1+/HLA-A1+* melanoma cells, indicating that the phage library-derived Fabs could recognize the native complex displayed on the surface of tumor cells. Compared to mouse hybridoma technology, the phage library screening is structure-dependent, fast, and cost-effective. This technique was subsequently explored in the study of TCR-like antibodies against peptide/MHC complexes derived from other tumor antigens, such as telomerase catalytic subunit [27], glycoprotein 100 (gp100) [23, 24], mucin 1 (MUC1) [28], human telomerase reverse transcriptase (hTERT) [27], NYESO-1 [29], MART-1 [34], preferentially expressed antigen in melanoma (PRAME) [45], tyrosinase [38], and WT1 [15]. TCR-like antibodies targeting virus epitopes derived from HTLV [46, 47], influenza [48], HIV [50, 52], and CMV [53] were also developed through the phage library strategy.

Early studies of these phage library-derived Fab antibodies focused on the use of antibodies as tools to detect the expression levels of peptide/MHC complexes on the tumor cell surfaces. To develop therapeutic strategies with phage library-derived TCR-like antibodies, researchers have used the CAR strategy by ligating the heavy chain variable (VH) and light chain variable (VL) region of the phage library-derived Fab antibody with the intracellular domain of CD3 molecules. The first TCR-like CAR-T strategy was developed in 2001 by ligating the VH and VL of the Fab antibody targeting the melanoma cells expressing MAGE-A1 and HLA-A1 [21]. The Fab recognizing the MAGE-A1 EADPTGHSY peptide/MHC complex on the melanoma cell surface was fused to the Fc (epsilon)RI-gamma molecule and retrovirally transduced into normal T cells. The transduced primary human T lymphocytes bound to the MAGE-A1 peptide/MHC complexes and responded to native *MAGE-A1+/HLA-A1+* target cells by specific cytokine production of interferon gamma (IFNγ) and tumor necrosis factor alpha (TNFα). These T cells could also lyse *MAGE-A1+/HLA-A1+* target cells but not control *MAGE-A1-/HLA-A1+* or *MAGE-A1+/HLA-A1-* tumor cells, indicating that the lysis of tumor cells via TCR-like antibodies was HLA-restricted and antigen-dependent. In a later study, the phage library-purified antibodies were further mutated through a combination of light (L) chain shuffling, heavy (H) chain-targeted mutagenesis, and in vitro selection of phage display libraries to be higher affinity (Fab-Hyb3) [22]. A functional study of Fab-Hyb3 found that the mutated TCR-like CAR-T mediated better recognition of the antigen on the tumor cell surface, indicating that the affinity of TCR-like antibodies dramatically affected the killing ability of the antibodies. The CAR-T technology has since been employed in several other TCR-like antibody studies, including those of gp100, minor histocompatibility antigen 1H (HA-1H), and WT1 [11, 25, 43, 44].

In 2006, Wittman and colleagues started to use the TCR-like antibody as a typical antibody therapy to mediate ADCC and CDC effects against tumors [31]. To target an HLA-A2-restricted peptide derived from human chorionic gonadotropin beta (hCG-β), which is overexpressed in over 90% of breast cancers, they developed a mouse IgG2a mAb (termed 3.2G1) via the hybridoma technique. The 3.2G1 antibody recognized the GVLPALPQV peptide from hCG-β presented by the HLA-A2 molecule and specifically stained the cells in a peptide- and antibody concentration-dependent manner. Staining of human tumor lines with the 3.2G1 TCR-like antibody also demonstrated the antibody’s ability to recognize endogenously processed peptides from the breast cancer cell line MDA-MB-231. Moreover, 3.2G1 antibody mediated CDC and ADCC against the human breast carcinoma MDA-MB-231 cell line in vitro and inhibited tumor implantation and growth in nude mice. These results provided valid evidence for the development of novel therapeutic antibodies that specifically kill tumors via recognition of peptide/MHC complexes. Since then, several TCR-like antibodies have been developed via the hybridoma strategy to mediate ADCC, CDC, or ADCP effects against tumor cells. These include TCR-like antibodies targeting peptide/MHC complexes derived from tumor protein 53 (TP53) [36], macrophage migration inhibitory factor (MIF) [40], proteinase 3 (PR1) [41], and WT1 [15, 44]. In addition to ADCC and CDC effects, the mouse hybridoma-derived TCR-like antibodies can also be utilized therapeutically to detect the expression of peptide/MHC complexes on the tumor cell surface as phage library-derived Fab antibodies [30, 32, 33, 37, 49, 51].

Because antibodies can be conjugated with toxins to deliver specific cytotoxic effects into cells, Denkberg and colleagues generated a conjugation molecule with a TCR-like antibody in 2003 [16]. In their study, a single-chain HLA-A2 molecule complexed with a common antigenic T cell HLA-A2-restricted epitope derived from the gp100 was used to immunize HLA-A2 transgenic mice. A phage display library was constructed from the immunized mice, and a recombinant single-chain fragment variable (scFv) antibody that could bind to the gp100 IMDQVPFSV peptide/MHC complex with a high affinity in the nanomolar range was isolated. When fused to a very potent cytotoxic effector molecule in the form of a truncated bacterial toxin, the TCR-like antibody could specifically kill antigen-presenting cells (APCs) in a peptide-dependent manner. In 2008, Epel and colleagues employed the same technology to fuse a truncated form of Pseudomonas exotoxin A with the phage-derived TCR-like antibody that specifically targets the FLRNFSLML peptide/HLA-A2 complex derived from TCR gamma alternate reading frame protein (TARP) [35]. The fusion molecule exhibited specific cytotoxic activity on breast and prostate cancer cells that correlated with their TARP and HLA expression patterns and inhibited the growth of human breast tumor cells in nude mice. These results demonstrated the power of the TCR-like antibody conjugation approach to generate novel targeting molecules to eliminate tumor cells with the unique specificity observed in cytotoxic CD8+ T cells [101]. In the same year, a TCR-like antibody targeting MART-1 conjugated with immunotoxin was also developed for anti-melanoma therapy [26].

TCR-like antibodies can also induce tumor cell death directly after binding to the peptide/MHC complex on the tumor cell surface [102]. In 2006, Verma and colleagues generated two TCR-like antibodies (RL4B and RL6A) that recognized peptides derived from hCG-β and human p68 RNA helicase. They found that two TCR-like antibodies destroyed tumor cells independently of immune effector mechanisms, such as ADCC and CDC. TCR-like antibodies mediated the apoptosis of tumor cells through selective and specific binding to p68 RNA helicase YLLPAIVHI and hCG-β GVLPALPQV peptide/HLA class I complexes, which triggered the activation of c-Jun N-terminal kinases (JNKs) and intrinsic caspase pathways. This signaling was accompanied by the release of mitochondrial cytochrome c and apoptosis-inducing factor. The apoptosis induced by the TCR-like antibodies was completely inhibited by soluble MHC tetramers loaded with relevant peptides and by inhibitors for JNKs and caspases. Thus, their study suggested the existence of a novel mechanism of TCR-like antibodies in the mediation of tumor cell destruction, in addition to ADCC and CDC. This mechanism would appear to be especially important due to the absence or tolerance of immune cells in cancer patients [103–105].

The major functions of TCR-like antibodies include the detection of peptide/MHC complexes, CAR-T strategy, ADCC, CDC, ADCP, immunotoxin targeting, and direct induction of tumor cell death. TCR-like antibodies can also be used as a block to prevent the recognition of normal tissue cells by self-reacting T cells in autoimmune diseases. In an experimental allergic encephalomyelitis mouse model, Aharoni and colleagues developed several monoclonal antibodies that bound to the complex of myelin basic protein (BP) peptide on mouse I-As [106]. The antibodies blocked the proliferative response of in vitro cultured T cells to the BP peptide/I-As complex without affecting the T cell response to an irrelevant peptide derivative from tuberculin on the same allele. The antibodies also inhibited experimental allergic encephalomyelitis in H-2s mice. Hence, antibodies directed specifically to the autoantigen/MHC complex may offer a highly selective and effective treatment in autoimmune diseases. Moreover, in 2004, Held and colleagues generated a high-affinity (Kd = 60 nM) antibody that specifically recognized the NY-ESO-1 (157–165) but not NY-ESO-1 (157–167) or a cryptic NY-ESO-1 (159–167) peptide/HLA-A2 complex. In a dose-dependent manner, the antibody blocked the recognition of NY-ESO-1/HLA-A2-positive tumor cells by NY-ESO-1 (157–165) peptide-specific CD8+ T cells [29].

### Molecular mechanisms of TCR-like antibodies against tumor cells

**Fig. 2:**
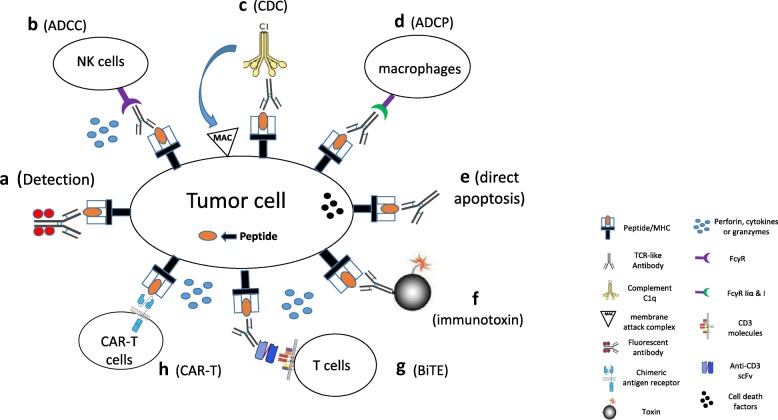
The molecular mechanisms of TCR-like antibodies against tumor cells. TCR-like antibodies mediate their anti-tumor effects through multiple mechanisms. (**a**) Conjugation with fluorescent reagents to detect the expression of the peptide/MHC complex on the surface of tumor cells. (**b**) Antibody-dependent cell-mediated cytotoxicity (ADCC) with NK cells. (**c**) Complement-dependent cytotoxicity (CDC) with complements. (**d**) Antibody-dependent cellular phagocytosis (ADCP) with microphages. (**e**) Direct induction of tumor cell apoptosis. (**f**) Conjugation with drugs or toxins. (**g**) Conjugation as a bi-specific T cell engager (BiTE). (**h**) CAR-T strategy. TCR-like: T cell receptor-like; MHC: major histocompatibility complex; MAC: membrane attack complex; FcγR: Fc gamma receptor; FcγR IIα: Fc gamma receptor II alpha

Due to the clinical prevalence of cancers, most studies of TCR-like antibodies have been conducted in the field of cancers. The major functions of TCR-like antibodies have been explored in two areas—the detection and measurement of the expression of tumor-specific peptide/MHC complexes on the tumor cell surfaces and the mediation of cytotoxicity against tumor cells. The detailed molecular mechanisms of TCR-like antibodies are summarized as follows (Fig. 2):

- TCR-like antibodies can be conjugated with fluorescent reagents to measure the expression levels of peptide/MHC complexes on tumor cell surfaces. By using a TCR-like antibody directed against the mucin epitope (HLA-A2-MUC1-D6) with calibration beads, Cohen and colleagues quantified the number of MUC1-D6-derived peptide/MHC complexes on the surface of tumor cells to be several hundred per cell [28]. Using TCR-like antibodies against peptide/MHC complexes of three major melanocyte differentiation antigens (gp100, Mart-1, and tyrosinase), Yael and colleagues found that melanoma cell lines had an average of approximately 4000 surface copies per cell of the complexes of HLA-A2 and tyrosinase (369–377) but only a few hundred copies per cell of gp100 and Mart-1 complexes [38]. This information about the antigen expression hierarchy is highly valuable when considering immunotherapy targets, because the levels of specific peptide/MHC complexes on tumor cells correlate with their susceptibility to cytotoxic T lymphocyte (CTL) killing [33].
- TCR-like antibodies can be converted to a CAR structure to mediate specific tumor lysis by T cells. The VH and VL regions of TCR-like antibodies can be converted to scFv and ligated to the intracellular domain of CD3 molecules. After being lentivirally or retrovirally transduced into patients’ T cells, the scFv region of TCR-like CAR can bind to the peptide/MHC complex on the surfaces of tumor cells. The intracellular domain of the TCR-like CAR can activate multiple cellular signaling pathways that lead to T cell activation and differentiation and secretion of cytokines, perforin, and granzymes [20, 27]. Many TCR-like antibodies isolated from phage display libraries that are in a monovalent antibody form (Fab or scFv fragments) have been successfully converted to CAR structures, and the TCR-like antibody derived from mouse hybridoma can also be converted [42].
- TCR-like antibodies can be used as a standard antibody therapy against tumor cells through ADCC, CDC, or ADCP. In ADCC, the full-length TCR-like antibodies, after binding to the peptide/MHC complex with Fab region, can bind to the Fc gamma receptors (FcγR) expressed on the surfaces of natural killer (NK) cells, monocytes, macrophages, neutrophils, eosinophils, and dendritic cells. These cells can then be activated to release cytokines and cytotoxic granules or express cell death-inducing molecules [31]. In CDC, the complement component 1q (C1q) binds to the TCR-like antibody and triggers the complement cascade. This leads to the formation of the membrane attack complex (MAC) (C5b to C9) at the surface of the target cells by the classical pathway of complement activation [41]. In ADCP, the TCR-like antibodies engage the Fc gamma receptors IIα (FcγRIIa) and FcγRI expressed on macrophages to trigger a signaling cascade that leads to the engulfment of the tumor cells [36].
- TCR-like antibodies can be conjugated with toxins or drugs to specifically lyse tumor cells [16, 35]. After the antibodies bind to cell surface antigens, some tumor cells can engulf the antibodies into the cytoplasm through the process of endocytosis. Diphtheria toxin (DT) or Pseudomonas exotoxin A are the most popular immunotoxins being evaluated in clinical trials because these bacterial toxins are easily produced in *E. coli* with high activity and few side effects in humans [107]. After being translocated to the cytosol, these toxins can catalyze adenine diphosphate (ADP) ribosylation of a diphthamide residue of elongation factor 2 (EF2), causing arrest of protein synthesis that leads to apoptotic cell death [108].
- TCR-like antibodies can be conjugated with secondary antibodies to specifically activate immune cells. The most commonly used secondary antibody is anti-CD3 scFv antibody, which can activate immune cells in a non-specific way. After binding to the peptide/MHC complex on the tumor cell surfaces, the anti-CD3 scFv-conjugated TCR-like antibodies recruit and activate immune cells to secrete perforin, cytokines, and granzymes at the proximity of tumor cells, leading to lysis of the tumor cells [17].
- TCR-like antibodies can induce tumor cell death directly after binding to the peptide/MHC complex. This effect is mediated by the activation of JNKs and intrinsic caspase pathways, accompanied by the release of mitochondrial cytochrome c and apoptosis-inducing factor in tumor cells [102]. Interestingly, this effect is not observed with pan anti-HLA antibodies lacking peptide specificity.

It is generally believed that, because of the repetitive antigen stimulation and in vivo selection process of hybridoma technology, TCR-like antibodies isolated using this technology have relatively high binding affinity (low nanomolar range) compared with the moderate to average binding affinity (~ 50–300 nM) of phage-derived TCR-like antibodies [31, 41, 53]. However, phage library-derived TCR-like antibodies of high affinity in the low nanomolar range have also been successfully isolated from second-generation libraries and by in vitro affinity maturation [22, 109]. TCR-like antibodies derived from both technologies have been evaluated in pre-clinical studies.

### Advantages and disadvantages of TCR-like antibodies versus other immunotherapies

The greatest advantage of TCR-like antibodies is their ability to target intracellular tumor antigens with minimal in vitro manipulation. The TCR-T adoptive cell therapy can also target intracellular antigens but requires a much more complicated preparation process [78]. In the traditional TCR-T adoptive cell therapy, the peripheral blood or tumor infiltration lymphocytes from a cancer patient must be isolated by apheresis. The lymphocytes are activated for 1 to 3 days to be transduced by TCR-containing lentivirus, retrovirus, or transposon vectors. The transduced T cells are then expanded to a large number (1 × 10^9^) before infusion back into the patient. The entire procedure takes about 3 to 4 weeks and is technically demanding, expensive, and time-consuming without the guarantee of success [94]. In addition, the transduced antigen-specific TCRs may mismatch with endogenous wild-type TCRs, as both TCRs exist in the same T cells [110, 111]. TCR-like antibodies, however, are relatively easy to prepare and store and used as off-the-shelf. Through the binding of the Fab region to the peptide/MHC complex, the Fc region of the TCR-like antibody can bind to the Fc gamma receptors (FcγR) expressed by patients’ NK cells, monocytes, or macrophage cells and activate these cells to kill tumors.

CAR-T is a specific form of tumor immunotherapy that equips the T cells with the tumor surface antigen-specific antibody and CD3 signaling pathway [112]. The recognition of tumor surface antigen by the antibody can trigger the CAR-T cell activation and the killing of tumor cells. The clinical success of CD19 CAR-T cells has proved their dramatic effect against tumors [113–115]. There are several reports of converting the TCR-like antibodies, especially the phage library-derived Fab antibodies, into CAR vectors [11, 25, 43]. T cells transduced with TCR-like antibody-derived CARs can specifically lyse tumor cells, indicating the therapeutic effectiveness of TCR-like antibody CAR-T cell therapy. Because of the lack of tumor-specific biomarkers on the surface of tumor cells, the traditional CAR-T therapy has achieved little success in solid tumors [116]. We envision that the TCR-like antibody CAR-T cell therapy could have specific value for solid tumors, as it targets intracellular tumor-specific antigens.

The checkpoint antibody strategy is a significant step in the history of humanity’s fight against cancer [117]. The molecular mechanism of this strategy is that the checkpoint antibody can reverse the immune suppression of tumor antigen-specific T cells that pre-exist in the patient’s body so that they may target the cancer cells [103]. The success of CTLA-4 and PD-1 checkpoint antibody therapy in the clinic has confirmed this mechanism [118]. However, checkpoint antibody therapy is effective in only about 20–30% of patients when used individually and 40–60% of patients when used in combination [119, 120]. These low rates indicate there may be a lack of tumor antigen-specific T cells at the tumor site, which hampers the effect of the therapy. TCR-like antibody therapy, however, does not depend on the existence of tumor antigen-specific T cells in the patient’s body and can activate the normal immune cells to target the tumor cells through ADCC, CDC, or ADCP [7, 18]. Combining TCR-like antibodies with checkpoint antibodies in future clinical studies may further improve the responses of patients.

Vaccine therapy is a longtime developed idea in the field of cancer immunotherapy, preceding the CAR-T cellular therapy and checkpoint antibody therapy [121]. The concept of using the host’s own immunity to fight cancers in the long-term has attracted significant interest from the scientific community. However, only two vaccines have currently been approved to treat cancer patients, and most tumor vaccines have shown poor clinical results, leading to their failure to secure approval from the US Food and Drug Administration (FDA) [122, 123]. It is hypothesized that the effect of a tumor vaccine is dependent on the development of memory immunity of tumor-specific T cells, and the tumor environment is usually plagued with immune-suppressive molecules [124]. Thus, it is difficult to induce a strong anti-tumor effect by the vaccine strategy. Moreover, the vaccine strategy is time-consuming and may take several months to develop tumor antigen-specific T cells. TCR-like antibodies, however, do not depend on the existence of tumor antigen-specific T cells and can take effect immediately after administration.

### The future of TCR-like antibody therapy

TCR-like antibodies, as new tools in the cancer immunotherapy field, have just begun to attract attention from the scientific community. By combining their fine specificity to recognize the peptide/MHC complexes of T cells with the biological and pharmacological properties of an antibody, TCR-like antibodies may have broad applications in the clinic. However, there are also several hurdles that must be overcome to achieve clinical success with the TCR-like antibodies.

First, TCR-like antibodies are MHC-restricted, which means that they are effective only for a certain group of patients expressing the tumor-specific antigen on a specific MHC allele. With HLA-A2 as the most common MHC allele in cancer patients, many tumor-specific peptides associated with this allele have been found [91]. Other HLA alleles, however, still lack tumor-specific peptides, which hamper the development of TCR-like antibody therapy. Further identification of less-common MHC-associated peptides will help solve this problem.

Second, the downregulation or absence of peptide/MHC complexes on the tumor cell surface is a common mechanism of tumor cells to evade immune surveillance [125]. TCR-like antibodies, like TCR-T therapy, may suffer from this effect. However, reports showed that some chemicals, cytokines, or radiation therapy can upregulate the expression of MHC and activate the MHC signaling pathway [126, 127]. Thus, TCR-like antibodies may combine with other therapies to achieve the best results. Furthermore, the affinity of TCR-like antibodies is generally higher than the affinity of in vitro synthesized TCRs [43]. The affinity of TCR-like antibodies can also be easily mutated to a higher affinity via molecular technology [22]. This will render antibodies more capable of recognizing the peptide/MHC molecule at extremely low levels.

Third, the immune-suppressive environment is a hurdle for the TCR-like antibody immunotherapy. Tumor cells reside in hidden sites to prevent the access of T cells, generate a hypoxic environment, and secrete a large amount of immune-suppressive cytokines, such as interleukin 10 (IL-10), transforming growth factor beta (TGF-β), or other molecules that cause the T cells, NK cells, macrophages, or monocyte to experience anergy or death [124, 128]. In addition, there are many suppressive immune cells around the tumor cells, which dampen the anti-tumor immune response [129, 130]. Thus, TCR-like antibodies may bind to the peptide/MHC complex on the tumor cell surface but might not mediate tumor destruction. Combining the TCR-like antibody therapy with other immune suppression-reversion therapy could help solve this problem. Examples may include the adoptive transfer of freshly expanded NK cells, monocytes, or macrophages in combination with TCR-like antibody therapy, or the combination of anti-PD-1 or anti-CTLA-4 antibody therapy. One advantage of TCR-like antibodies is that they can easily penetrate the tumor environment and they do not require the existence of tumor antigen-specific T cells at the tumor site. Furthermore, some of the TCR-like antibodies can induce tumor cell death directly through binding to the peptide/MHC complex [39, 102].

## Conclusion

With only a few dozen TCR-like antibodies reported in publications, we have limited knowledge about this new group of antibodies. However, with solid evidence of their effectiveness in hematological and non-hematological preclinical tumor models and unique character to detect the expression levels of tumor-specific peptide/MHC complexes on the surface of tumor cells, TCR-like antibodies may represent an ideal next step for cancer immunotherapy.

## Data Availability

The dataset supporting the conclusions of this article is included within the article.

## Abbreviations

ABL: Abelson murine leukemia viral oncogene homolog 1
ADC: Antibody-drug conjugate
ADCC: Antibody-dependent cellular cytotoxicity
Adcetris: Brentuximab vedotin, abti-CD30 antibody-drug conjugate
ADCP: Antibody-dependent cellular phagocytosis
APCs: Antigen-presenting cells
ATL: Adult T cell leukemia
BCR: B cell receptor
BiTEs: Bi-specific T cell engaging antibodies
BL: Burkitt’s lymphoma
BP: Basic protein from myelin
C1q: Complement component 1q
CAR: Chimeric antigen receptor
CAR-T: Chimeric antigen receptor T cells
CD19: Cluster of differentiation 19
CD20: Cluster of differentiation 20
CD3 ζ: CD3 zeta
CDC: Complement-dependent cytotoxicity
CEA: Carcinoembryonic antigen
CMV: Cytomegalovirus
CTLA-4: Cytotoxic T-lymphocyte-associated protein-4
DT: Diphtheria toxin
E5: Papillomavirus E5 antigen
E6: Papillomavirus E6 antigen
E7: Papillomavirus E7 antigen
EBNA: Epstein-Barr nuclear antigen
EbV: Epstein-Barr virus
ER: Endoplasmic reticulum
F293I: Phenylalanine mutated to isoleucine at 293 position
Fab: Fragment antigen-binding
FC: Fragment crystallizable region
FcγR IIα: Fc gamma receptor II alpha
FcγR: Fc gamma receptor
FDA: US Food and Drug Administration
G12D: Glycine mutated to aspartic acid at 12 position
H chain: Immunoglobulin heavy chain
HA: Viral hemagglutinin
HBV: Hepatitis B virus
HBX: Hepatitis B virus X protein
HCC: Hepatocellular carcinoma
HCV: Hepatitis C virus
HER2: Human epidermal growth factor receptor 2
HIV: Human immunodeficiency virus
HIV.Gag: Group-specific antigen from HIV
HIV-Pol468: Pol reading frame 468 from HIV
HLA: Human leukocyte antigen
HPV: Human papillomavirus
hsp70-2: Heat shock protein 70-2
hTERT: Telomerase reverse transcriptase
HTLV: Human T cell leukemia-lymphoma virus
I-As: Mouse MHC allele
IFN-γ: Interferon gamma
JNK: C-Jun N-terminal kinases
K122N: Lysine mutated to asparagine at 122 position
Kadcyla: Ado-trastuzumab emtansine, anti-HER2 antibody-drug conjugate
Keytruda: Pembrolizumab, anti-PD-1
Kras: Kirsten rat sarcoma virus oncogene
KS: Kaposi’s sarcoma
KSHV: Kaposi sarcoma-associated herpesvirus
Kymriah: Tisagenlecleucel, anti-CD19 CAR-T cells
L chain: Immunoglobulin light chain
L265P: Leucine mutated to proline at 265 position
LANA: Virus latency-associated nuclear antigen from KSHV
LMP1: Latent membrane protein 1
LMP2: Latent membrane protein 2
mAb: Monoclonal antibody
MAC: Membrane attack complex
MAGE: Melanoma-associated antigen
MART: Melanoma antigen recognized by T cells
MCC: Merkel cell carcinoma
MCMV: Murine cytomegalovirus
MHC: Major histocompatibility complex
MYD88: Myeloid differentiation primary response 88
Mylotarg: Gemtuzumab ozogamicin, anti-CD33 antibody-drug conjugate
NHL: Non-Hodgkin’s lymphoma
NK: Natural killer
NP: Nucleoprotein
NPC: Nasopharyngeal carcinoma
NS3: Virus nonstructural protein 3 from HCV
NS5A: Virus nonstructural protein 5A from HCV
NYESO-1: New York esophageal squamous cell carcinoma-1
Opdivo: Nivolumab, anti-PD-1
OVA: Ovalbumin
PD-1: Programmed cell death receptor-1
PEL: Primary effusion lymphoma
PP65: CMV PP65 antigen
PRAME: Preferentially expressed antigen in melanoma
S37F: Serine mutated to phenylalanine at 37 position
scFv: Single-chain fragment variable
SSX: Synovial sarcoma X
SV40: Simian virus 40
TARP: TCR gamma alternate reading frame protein
Tax: The viral transactivator
TCR: T cell receptor
TNFα: Tumor necrosis factor alpha
TP53: Tumor protein p53
vFLIP: Virus FLICE/caspase-8-inhibitory protein from KSHV
vGPCR: Virus active G protein-coupled receptor homolog from KSHV
VH: Heavy chain variable
vIRF-1: Virus IFN-inducible factor from KSHV
VL: Light chain variable
VSV: Vesicular stomatic virus
WT-1: Wilms tumor gene-1
Yervoy: Ipilimumab, anti-CTLA-4
